# Nutritional conditions in oral squamous cell carcinoma patients –prospective longitudinal observational study

**DOI:** 10.1186/s13005-026-00604-2

**Published:** 2026-03-07

**Authors:** Caroline Wurche, Julia Wirth, Johannes Kleinheinz, Lauren Bohner, Sonja Sielker

**Affiliations:** 1 Department of Cranio-Maxillofacial Surgery, Research Unit Vascular Biology of Oral Structures (VABOS), University Hospital Muenster, Waldeyerstraße 30, D-48149 Muenster, Germany; 2 Department of Dentistry, Federal University of Santa Catarina, Florianópolis, Brazil

**Keywords:** Malnutrition, OSCC, Nutritional status, Nutritional treatment, Cancer-related cachexia

## Abstract

**Background:**

Cancer-related malnutrition is an often-disregarded problem with a lack of knowledge and standards concerning diagnosis and therapy. Patients with oral squamous cell cancer can suffer not only from metabolic factors but also from aggravated oral food intake. This study examines the issue of malnutrition in patients with oral squamous cell carcinoma (OSCC) before diagnosis and under treatment.

**Methods:**

The nutritional status of 30 patients with OSCC was registered before and after surgical treatment, with a time interval of 16 weeks. The methods applied were body mass index (BMI), subjective global assessment (SGA) and bioelectrical impedance analysis (BIA). The reference group consisted of 30 patients planned for orthognathic surgery. Equal for both groups were postoperative nasogastric tube feeding. The data for the OSCC patients were analysed and tested for significance at the level of *p* = 0.05.

**Results:**

Preoperative malnutrition, as expected, was only detected in the OSCC group. Only 6% of OSCC patients start with a good nutritional status, but this increases to nearly 50% during therapy, although two patients ended up with severe malnutrition. Nonetheless, 40% of the reference group were malnourished after the first week, according to the SGA. The nutritional status improved during therapy to a good status in nearly all cases.

**Conclusion:**

Malnutrition is a relevant problem in patients with OSCC due to the high prevalence in this population, which was confirmed by these data. Appropriate diagnostic tools and an adapted treatment concept in terms of application form and ingredients, as well as supplements, should be an integral part of the treatment concept for these patients. Additionally, the data from these studies recommend that patients who are preoperatively healthy and have temporary postoperative restrictions on oral food intake be provided with an adequate nutrient supply. An important component of nutritional interventions should be the long-term prevention of skeletal muscle loss.

## Background

The treatment of cancer patients usually includes multimodal concepts, with the implementation of various disciplines in interdisciplinary teams. Even if the prevalence and consequences of cancer-related cachexia are well known, the problem of malnutrition is often disregarded by all involved participants. Hence, patients are left alone and may seek questionable so-called cancer diets via the internet.

At the time of diagnosis, already 54% of all cancer patients notice a loss of weight and more than 25% of all cancer patients die only due to the malnutrition [1–3]. There is a lack of knowledge and standards in the diagnosis of malnutrition and clinical nutrition in all professions. This issue is just considered to be facultative instead of a mandatory part of the therapy, with no concrete assignment of responsibility to any profession [3–6].

The causal of this issue is aggravated oral food intake, especially in the case of oral squamous cancer (OSCC). However, in addition, for all oncological diseases, cancer-related multifactorial changes can be summarized as cancer-related cachexia. This term includes the metabolic shift due to the cancer-related inflammation with the loss of fat and muscle tissue and the reduced food intake because of physical and mental stress. Further, the term includes hyper-catabolism with insulin resistance, hyperglycaemia, protein loss, anaemia, fatigue, and infection susceptibility [2, 7, 8]. This creates a vicious circle of reduced mobility, reduced power, and lethargy with a loss quality of life, complications, and, ultimately, morbidity and mortality [2, 9–13]. Consequently, malnutrition in the OSCC group results in increased rates of pneumonia, surgical site infections, and, ultimately, increased mortality [14–17]. In the treatment of the OSCC, malnutrition is mentioned in the current German guidelines. On the one hand, patients should receive a professional nutritional therapy in the early stage. Otherwise, no tools or standards are recommended–diagnosing or conducting this nutritional therapy. The different diagnostic methods and definitions make it difficult to compare data and lead to uncertainty in practice [8, 13, 16, 18, 19].

Nevertheless, different definitions and measuring methods has been published to diagnosing a cancer-related malnutrition. One method for describing the nutritional status of patients is to determine body weight and, for easy adjustment, to calculate the body mass index (BMI) as the ratio of body weight to height squared. The BMI, or generally weight control, as a simple and easy workable technique, is a good part for monitoring the nutritional status. For further adjustments, there are tables with age, sex or different classifications of the nutritional status such as underweight, healthy, overweight or obesity. Underweight, according to the World Health Organization (WHO) is defined as having a BMI below 18.5 kg/m² [20].

This should be extended to include the aetiology. A classification is provided by the guidelines of the European Society for Clinical Nutrition and Metabolism (ESPEN), in which the term cancer-related cachexia is found, subsumed in the category of disease-related malnutrition. The definition of Fearon is used here, which has been expanded to include the following criteria: weight loss > 5% over the past 6 months or BMI below 20 kg/m² and any degree of weight loss > 2%; or appendicular skeletal muscle index consistent with sarcopenia (males < 7–26 kg/m²; females < 5–45 kg/m²) and any degree of weight loss > 2% [8]. The American Society of Parenteral and Enteral Nutrition (ASPEN) uses 6 diagnostic criteria: low-energy intake, weight loss, loss of muscle mass, loss of subcutaneous fat, fluid accumulation, and hand grip strength [21].

But there are other frequently used diagnostic methods with additional measuring methods. To enhance the unidimensional parameter of BMI and include more anamnesis and clinical examination, other assessments have been developed and are recommended [13]. An established, often used and therefore comparable example is the subjective global assessment (SGA) by Detsky [22]. The SGA is separated into three sections: the medical history, the physical examination with a focus on malnutrition-specific changes, such as the measurement of subcutaneous adipose tissue, muscle atrophy or oedema in defined areas of the body. Finally, a subjective assessment of the nutritional status by a qualified examiner. The focus lies in weight loss, dietary intake change, loss of subcutaneous fat tissue, and muscle atrophy. This results in a three-level categorization of good (category A), moderately malnourished (category B) or severe malnourished (category C) [22].

Another tool for determining nutritional status with the advantage of providing objective and comparable data is the bioelectrical impedance analysis (BIA). The BIA measures the different electrical conductivities of tissues and therefore reveals changes of the body compartments with the possibility for further description of malnutrition. The standard procedure to determine the individual body compartments is based on the three components: total body water, body fat, and lean body mass (LBM). The lean body mass is subdivided into the body cell mass (BCM) and the extracellular mass (ECM). This method is based on the different electrical conductivity of different tissues. With the loss of skeletal muscles and body fat, the BCM decreases while the ECM increases, and the LBM remains constant. Especially, the loss of muscle causes a shift in intracellular body water to extracellular, which strongly distinguishes tumour-related cachexia from starvation metabolism [23–25].

There are various values discussed in the literature for the constellation of the relevant parameters and their cut-off values; we decided in favour of the standard values specified by the manufacturer of the NutriBox (Data Input GmbH, Pöcking, Germany) [25, 26].

Despite the severe consequences of malnutrition in cancer patients, and OSCC patients in particular, there is a scarcity of data on its prevalence and progress following surgical treatment. Therefore, this should be the aim in this pilot study. By comparing the BMI, the SGA score and the BIA measurement method, the best and practicable instrument for initial diagnosis and follow-up should be determined. Patients undergoing orthognathic surgery are employed as a reference group due to the absence of data regarding healthy patients without systemic diseases that affect nutritional status but who have postoperative restrictions on food intake and are receiving temporary nasogastric feeding. This should enable comparisons between changes due to systemic cancer-related cachexia, rather than postoperative nutritional intake restrictions and postoperative tube feeding.

## Methods

### Study design and ethics approval

In the present study, the nutritional status of 30 patients with histological verified primary OSCC was determined during the cancer treatment and afterwards with a time interval of 4 months. All patients were first treated surgically and, if necessary, received adjuvant radio(chemo)therapy in accordance with the interdisciplinary tumour conference decision. As a reference group for standard nutritional values, 30 patients undergoing orthognathic surgery who also received a standardized nutrition via nasogastric tube during post-operatory period, were included. All patients received a ready-made, fully balanced, normocaloric standard tube feeding (Nutrison Multi Fiber, Nutricia, Danone Deutschland GmbH, Frankfurt, Germany) without adjustment to disease- or patient-specific characteristics such as increased amounts of calories, protein, or fibre. The daily dose was distributed over 3–4 applications using gravity systems, with the aim of covering the estimated calorie requirement based on body weight. This was adjusted according to the patient’s condition.

Included were patients with an age over 18 years, agreement to the study protocol, participation in examinations, and completed follow-up over 4 months. Excluded were patients with any other oncological diagnosis or relapse, also patients with gastrointestinal diseases such as chronic inflammatory bowel disease or gluten-related disorders. Further, pregnant women, person with a pacemaker, or an implanted cardioverter-defibrillator, or likewise devices. The study was designed according to the “Declaration of Helsinki” and approved by the Ethics Committee of the Faculty of Medicine, University of Muenster (#2018-642-f-S). A written informed consent was obtained from all patients.

### Body-mass-index (BMI)

The BMI was estimated with an electrical column scale (type 769, SECA, Hamburg, Germany). During measurement, the patient stands lightly dressed upright and flat on the scale. Patients were included in the BMI categories, according to the World Health Organization (WHO): mild thinness (< 18.5 kg/m^2^), normal range (18.5–24.9 kg/m^2^), overweight (25.0–29.9 kg/m^2^), obese I (30.0–34.9 kg/m^2^), obese II (35.0–39.9 kg/m^2^), and obese III (> 40 kg/m^2^) [20].

### Subjective global assessment (SGA)

The SGA was performed according to Detsky [22]. A well-trained examiner performed the examination steps. The SGA was separated into three parts: the anamnesis, a physical examination, and afterwards the subjective assessment about the nutritional status. The patients were grouped as A well nutritional status, B moderate malnourished, and C severe malnourished [17, 18, 21, 23]. For grouping, no weighted scoring system was used. Patients were classified as moderately malnourished, when patients lost 5% of body weight without any earlier recognized stabilizations nor weight gain and in addition, a loss in food supply and a mild subcutaneous tissue loss. Patients severe malnourished, when patients lost at least 10% of body weight, a severe loss of subcutaneous fat and muscle mass, and the occurrence of oedema.

### Bioelectrical impedance analysis (BIA)

The BIA was performed by a well-trained examiner according to manufacture protocols with the Bioelectrical Impedance Analyzation (NutriBox; Data Input GmbH, Pöcking, Germany). Before examination, patients remained fasting for at least four hours without being physically active for at least 12 h. The bladder was emptied before measurement. No alcohol was consumed for at least 24 h. For measurement, the patients lay flat and horizontal in the supine position on a non-conductive surface at complete physical rest. Electrodes were placed, according to manufacture protocols, on the hand and the foot of the dominant side of the body. Hand and foot were unclothed and free of compressive bandages. To ensure that the patient’s blood volume and body water were distributed evenly throughout the body, the patient remain in this position for nearly 10 min before the measurements starts, based on manufacturer’s protocols. According to manufacturer protocols, patients were categorized as malnourished if at least two of the three parameters, such as the phase angle (< 4°), ECM/BCM index (> 1), and % cell ratio (< 35% for men, < 30% for women), deviated from the norm.

### Statistical analysis

Statistical analysis was performed with the software SPSS Version 29 (IBM). All tests were performed considering a statistical significance level of *p* = 0.05. The variables dielectric phase angle, BMI, weight, and body fat were assessed as dependent quantitative variables. Time of follow-up was set as an independent categorical variable. Baseline values (T0) were assessed one day before surgery. As post-operative, the following follow-up times were considered: T1-one after surgery, T2- 2 weeks after surgery, T3- 4 weeks after surgery, T4- 8 weeks after surgery and T5- 16 weeks after surgery. First, descriptive data were graphically analysed comparing the OSCC group to the reference standard values. After, for OSCC group, changes in nutritional status over time were assessed and compared to the baseline values (T0).

ANOVA for repeated measures with Bonferroni adjustment was used to evaluate the mean difference of nutritional values over the time. A simple contrast was applied, considering the baseline values as reference. In addition, changes of phase angle values over time were assessed by means of a summary of measures and compared to the reference values. For both groups, a regression analysis was estimated for each patient, and the estimated slopes were used as outcomes. There was a difference on regression coefficient of tumor patients in comparison to the reference group (*p* = 0.004). Mean differences between groups were assessed by Mann-Whitney-U test.

## Results

Table 1 provides a summary of the comparative analysis of both groups. For OSCC group, the mean nutrition time via nasogastric tube was 16 days, with a hospital stay of 18.9 days (Table 1). In the OSCC group, complications were reported in 12 cases. In six cases, wound healing impairments occurred. Two patients suffered from dysphagia. In other cases, patients underwent a re-resection, a radial flap revision, atrial fibrillation and optic nerve ischaemia, respectively. In the reference group, a total of three patients showed a complication, with the development of a peri-mandibular abscess in two cases and paraesthesia in one case.

**Table 1:** Comparative overview about patients’ data. The numbers indicate the quantity of patients. The percentage or range is shown in brackets. The mean is indicated for all other groups, while the median is utilized for age

	reference group (*n* = 30)					OSCC group (*n* = 30)				
Patient data										
Sex	19 male / 11 female					23 male / 7 female				
Age (total)	25 years [19–60 years]					64 years [42–85 years]				
Nutrition tube (total)	2.9 days [1–5 days]					16 days [7–28 days]				
Hospital stays (total)	7.6 days [7–10 days]					18.9 [9-37days]				
Body mass index (BMI) categories										
Pre surgery patient [%]	mild thinness		1	[3.3%]		mild thinness		**--**		
	normal range		17	[56.7%]		normal range		6	[20%]	
	overweight		8	[26.7%]		overweight		15	[50%]	
	obese I		2	[6.6%]		obese I		9	[30%]	
	obese II		1	[3.3%]		obese II		**--**		
	obese III		1	[3.3%]		obese III		**--**		
Patients with a change in BMI categories after 12 weeks	5 [16.7%]					12 [40%]				
Number of patients with a loss of weight and weight reduction in kilograms (kg)										
During observation time [%]	27 [90%]					27 [90%]				
After 1 week (total)	3.8 kg (0.9–9.0 kg) in 28 patients					3.7 kg [0.3–7.1 kg] in 25 patients				
After 4 weeks (total)	5.0 kg [0.5–14.0 kg] in 29 patients					5.1 kg [0.3–10.8 kg] in 26 patients				
After 12 weeks (total)	4.3 kg [0.5–12.6 kg] in 27 patients					6.1 kg [0.1–16.1 kg] in 27 patients				
Number of patients within subjective global assessment (SGA) status groups										
	A status	B status			C status	A status	B status			C status
Pre surgery [%]	30 [100%]	**--**			**--**	6 [20%]	24 [80%]			**--**
After 1 week [%]	18 [20%]	12 [40%]			**--**	7 [23.3%]	23 [76.7%]			**--**
After 4 weeks [%]	16 [53.3%]	14 [46.7%]			**--**	9 [30%]	19 [63.3%]			2 [6.7%]
After 12 weeks [%]	25 [83.3%]	5 [16.7%]			**--**	14 [46.7%]	12 [40%]			4 [13.3%]

Based on the visual comparative analysis from the SGA, patients from the reference group were moderately malnourished during hospital stay. Nutritional status raised to a good status in nearly all cases. In contrast, only 6% of OSCC patients starts with a good nutritional status. During treatment time and afterwards, the ratio rose to nearly 50% of OSCC patients. However, two patients ended up with severe malnutrition (Table 1).

**Fig. 1:**
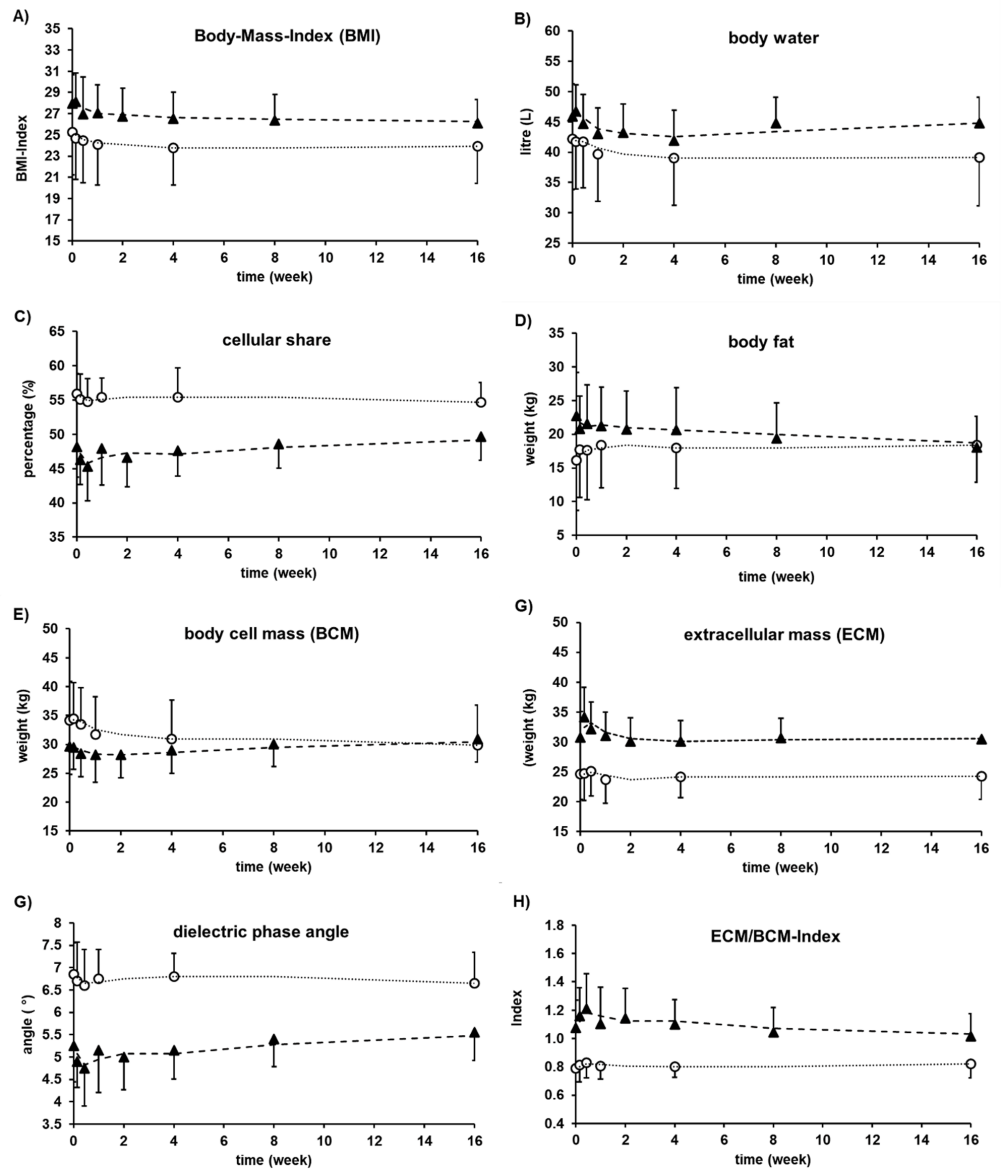
Summary of results received from BMI and from BIA examination. Shown is the median. For clear arrangement, examination “pre surgery day” was set as time point zero and error bars with one-site direction. (circles= reference group, triangle = OSCC group; trend lines = sliding average, dotted for reference group and dashed for OSCC group)

Figure 1 shows the results from BIA and BMI. In the OSCC group, BMI fell by 2 points over time. The BMI ranged between 24 and 26. In the reference group, the BMI remained constantly within a variation of 1.5 points. During the study period, the BMI values of all three patients changed. However, the change was not significant enough to alter their BMI category. The amount of body water sunk in both groups during the first days. Afterwards, the amount of body water increased in the OSCC group. In the reference group, the cellular share levels between 54.7% and 55.5%. Therefore, in the OSCC group, it increased from 46.3% to 49.7% continuously (Fig. 1C). The amount of body fat levels between 17.7 kg and 18.4 kg in the reference group and decreased constantly during the observation time from 20.8 kg to 18.0 kg in the OSCC group (Fig. 1D). In the OSCC group, the BCM decreased during the first weeks and increased afterwards continuously (Fig. 1E). On the other hand, the ECM decreased in this group constantly (Fig. 1F). The BCM/ECM index decreased to an end value of nearly one, accordingly (Fig. 1H). In the reference group, the BCM decreased over time and the ECM remained constant. The BCM/ECM index levels between the values of 0.8 for the whole examination period (Fig. 1H). For the dielectric phase angle, there was a comparable shift. In the reference group, the value levels between 6.78° and 6.6° during the examination period. In the OSCC group, the angle increased from 4.9° to 5.5° continuously (Fig. 1G).

Statistical analysis is summarized in Tables 2 and 3. Except for the BIA parameter body water (*p* = 0.12), there was a statistically significant decrease of phase angle, BMI, Weight, and fat in comparison to baseline values (Table 3). There was a difference on regression coefficient of OSCC group in comparison to the reference group (*p* = 0.004). Reference group presented a median (interquartile) from − 0.011 (0.03), whereas OSCC group showed a positive regression coefficient median (interquartile) from 0.010 (0.05).

**Table 2:** Descriptive data of nutritional change values and mean difference from post-operative measurements to the baseline (SD = standard deviation; * = means statistical significance level at *p* = 0.05)

value	Mean (SD)	95% CI (Inferior)	95% CI (Superior)	Mean difference to the baseline	*p*-value
Phase Angle					
Pre-operative (baseline)	5.35 (0.92)	5.01	5.70		
1 week	5.22 (1.30)	4.73	5.71	0.27*	0.008
2 weeks	5.08 (0.88)	4.75	5.41	0.27*	0.02
4 weeks	5.22 (0.83)	4.91	5.53	0.13	0.28
8 weeks	5.27 (0.82)	4.96	5.58	0.08	0.47
16 weeks	5.45 (0.82)	5.14	5.76	-0.09	0.41
BMI					
Pre-operative (baseline)	27.96 (3.42)	26.68	29.24		
1 week	27.08 (3.35)	25.82	28.33	0.883*	< 0.001
2 weeks	26.73 (3.34)	25.48	27.98	1.23*	< 0.001
4 weeks	26.54 (3.09)	25.38	27.69	1.42*	< 0.001
8 weeks	26.39 (2.90)	25.30	27.47	1.57*	< 0.001
16 weeks	26.12 (2.63)	25.14	27.10	1.84*	< 0.001
Weight					
Pre-operative (baseline)	83.95 (10.72)	79.94	87.95		
1 week	81.25 (10.13)	77.46	85.03	2.70*	< 0.001
2 weeks	80.18 (9.95)	76.46	83.89	3.77*	< 0.001
4 weeks	79.62 (9.27)	76.16	83.09	4.32*	< 0.001
8 weeks	79.12 (8.50)	75.94	82.29	4.83*	< 0.001
16 weeks	78.38 (7.45)	75.59	81.16	5.57*	< 0.001
Fat					
Pre-operative (baseline)	22.13 (7.55)	19.31	24.95		
1 week	21.25 (6.94)	18.65	23.84	0.88	1.00
2 weeks	20.97 (6.83)	18.41	23.52	1.16	1.00
4 weeks	20.10 (7.45)	17.32	22.88		
8 weeks	19.09 (6.23)	16.76	21.41		
16 weeks	18.24 (5.67)	16.12	20.36		
Water					
Pre-operative (baseline)	45.32 (7.14)	42.65	47.98		
1 week	43.89 (6.30)	41.54	46.24	1.43	0.433
2 weeks	43.84 (6.25)	41.50	46.17	1.48	0.937
4 weeks	43.66 (6.35)	41.29	46.04	1.65	0.373
8 weeks	43.94 (5.66)	41.82	46.05	1.38	0.627
16 weeks	44.02 (5.73)	41.88	46.16	1.29	0.831

**Table 3:** ANOVA for repeated measures

value	Sum of squares	df	Mean square	F-value	*p*-value
Phase angle	3.27	5	0.65	4.02	0.002
BMI	63.80	2.07	30.79	20.48	< 0.01
Weight	590.90	2.09	282.63	20.71	< 0.01
Fat	313.31	3.32	94.30	9.73	< 0.001
Water	54.48	3.13	17.38	1.91	0.12

## Discussion

In this study, we monitored changes in the nutritional status of OSCC patients before and after surgical treatment by BMI, SGA and BIA, with a time interval of 4 months.

In this study, the BMI showed no case of malnutrition (Fig. 1A) over the whole observation period. Unlike the SGA and the BIA, which showed prevalence’s of malnutrition in the study population with OSCC of 80% and 60%, respectively (Table 1; Fig. 1). Concerning malnutrition, BMI as a standalone parameter is far from sufficient according to this data because essential factors are not recognised. These data therefore underline the need for specialised assessments such as SGA or BIA [3].

The BMI’s inadmissibility was also seen in the reference group. Within the first 4 weeks postoperatively, however, nearly 50% of the patients switched according to the SGA to category B, i.e. were moderately malnourished. Similarly, BIA values have changed, while BMI categories have remained the same (Fig. 1). This study demonstrates that SGA and BIA, in contrast to BMI, exhibited a similar tendency and detected patients at risk of cancer-related cachexia and malnutrition after maxillofacial surgery more frequently.

The definition of a gold standard would be desirable to enable good comparability of the data, as different assessments are currently being made in different studies. There are no detailed data available with the criteria selected by Fearon for cancer cachexia [8].

For the OSCC group, this study observed that already about 6 months before treatment, 87% of OSCC patients reported a weight loss. This number appears to be very high compared to reported prevalence rates of weight loss, with 54% over all oncology patients 6 months before diagnosis [1–3]. The possible cause in OSCC patients could be impaired food intake due to local mechanical restrictions. A more pronounced inflammation with increased catabolism or comorbidities resulting from the risk factors are also conceivable causes [2, 7, 8]. This underlines the usefulness of the definition of Fearon and ESPEN to consider the anamnestic weight loss and emphasize the prevalence of this problem in patients with OSCC.

The limitations of the method due to the characteristics of the reference group in comparison to the OSCC should be emphasised. A generalisability of the statements from the OSCC in comparison to the reference group is clearly limited. The groups differ due to age, comorbidities, therapy received and, in particular, the different durations of tube feeding. The orthognathic reference group is expected to be younger and healthier due to higher incidence of dysgnathia in younger age having less comorbidities. This is proved in our data with a median age of 25 years comparable to 64 years in OSCC group (Table 1). Even though this study did not focus on comorbidities, a lower prevalence of compromising diseases within the reference group could be expected. Furthermore, there were significant differences in the duration of the tube feeding between the groups (Table 1). However, conclusions could still be gained from comparing these groups. So, both groups got the same standardized non-individualised dietetic treatment by a nutrition tube and suffered from postoperative restrictions on food intake.

In SGA and BIA, malnutrition was exclusively detected in the OSCC group preoperatively, as previously mentioned. This underscores the necessity of presuming that patients with OSCC are already malnourished before surgery. In contrast, limited oral food intake, e.g. in cases of dysgnathia, does not have the same impact on the nutritional status as in patients with cancer-related cachexia.

In this study, we could demonstrate that this unspecific dietetic treatment strongly influenced the nutritional status and alleviated malnutrition in OSCC patients. The nutritional status, as assessed by SGA and BIA, showed significant reductions under simple, non-individualised nutritional therapy utilising standardized nutrition via tube during postoperative hospitalisation. At the beginning of the study, 20% of OSCC patients showed an SGA category A and raised up to 50% of OSCC patients at the end of the study (Table 1). However, a small proportion of 13% of the OSCC collective (*n* = 4) also deteriorated during therapy and fell into the worst category C of the SGA, severe malnutrition (Table 1). This is suspected to be caused by the exhausting oncological therapy, psychological factors or morbidities due to surgery and possibly adjuvant radiotherapy. These factors should be considered in further studies.

The data from the BIA gave the most reliable information about shifts in nutritional status. In the OSCC group, this angle raised continuity to 5.5° during time (Fig. 1G). Further, the amount of body fat sank, and the amount of muscle mass raised simultaneously in this group. The BCM/ECM index sank to a value of nearly one, deductively (Fig. 1H). Caped all BIA values, a regeneration from malnutrition to a better nutritional status was observable in the OSCC group. The early changes in nutritional status could be explained by the surgery and circumstances itself, comparable to the reference group.

These similar results and developments in SGA and BIA mean that both methods can be regarded as suitable based on this data, whereas the cutoffs of the BIA should be reviewed. There is a certain range in the literature, which could be a reason for Barbosa-Silva to conclude that the two methods are less similar in their significance [27, 28].

Observing the development of the reference group, within the first 4 weeks postoperatively, nearly 50% of the reference group switched according to the SGA to category B, i.e. were moderately malnourished. At the end of the observation period after 4 months, 83.3% of the patients had recovered to category A (Table 1). Awareness of malnutrition in the context of such interventions seems to be limited to a few studies, often focusing only on weight, but showing similar tendencies [29–31].

This study showed how important diagnosis, monitoring, and therapy of malnutrition for patient’s outcome is. Not only concerning oncological patients, but also patients like the reference group with orthognathic surgery or potentially with any kind of head and neck surgery. In particular, change in oral food intake due to local restrictions and intermaxillary fixation should be considered. Furthermore, this simple, non- individualized standardised nutrition therapy could change hospital stay and nutritional status of oncological and orthognathic surgery patients. It can even be assumed that this should also be critical for other indication groups in the head and neck surgery.

This study provides the comparative data for further studies to investigate the effect of different nutritional therapies and the effect on various parameters such as BMI, SGA, BIA, and in perspective blood parameters concerning nutrition (e.g. albumin, LDL, HDL etc.). Furthermore, hospitalisation, prognosis, morbidity, mortality, quality of life, and patient-reported outcome should be investigated. Observational studies with professional individualized nutrition therapy should follow to verify these data. In a relevant proportion of OSCC patients over 80 years, data should also be collected with various groups of age to distinguish tumour-associated cachexia from frailty in the elderly.

The requirement is to use assessments to diagnose malnutrition early and establish a standardized follow-up to prevent its multiple consequences. This is already stipulated in the German guideline of OSCC, but concrete recommendations for implementing an assessment or nutritional concepts have yet to be established. Especially in the field of head and neck surgery, not only the form of application but also the composition should be taken into focus of all participants. This requires the awareness, cross-sectoral and interdisciplinary cooperation at all points of time from pre-habilitation to the follow-up.

## Conclusion

Malnutrition is a relevant problem in patients with OSCC, as the prevalence in this population group is very high. This was confirmed by our results. Appropriate valid diagnostic tools and an adapted individualized treatment concept should be an integral part of the treatment concept for these patients. Professional dietary treatment is necessary and should be provided from the time of diagnosis until aftercare. It is essential that patients are not left to deal with this issue alone. Especially in relation to certain dubious “cancer diets” that are available on the Internet. We demand a personalized concept to the form of application, ingredients, and supplements for each patient.

## Data Availability

The datasets analysed during the current study are available from the corresponding author on reasonable request.

## Abbreviations

ANOVA: Analysis of variance
ASPEN: The American society of parenteral and enteral nutrition
BCM: Body cell mass
BIA: Bioelectrical impedance analysis
BMI: Body mass index
ECM: Extracellular mass
ESPEN: The European society for clinical nutrition and metabolism
LBM: Lean body mass
LDL: Low density lipoprotein
HDL: High density lipoprotein
SGA: Subjective global assessment
WHO: World Health Organization

## References

[1] Dewys WD, Begg C, Lavin PT, Band PR, Bennett JM, Bertino JR, et al. Prognostic effect of weight loss prior to chemotherapy in cancer patients. Eastern Cooperative Oncology Group. Am J Med. 1980;69:491–7. 10.1016/s0149-2918(05)80001-3. 7424938 10.1016/s0149-2918(05)80001-3

[2] Arends J. Ernährung von Tumorpatienten. Aktuel Ernahrungsmed. 2012;37:91–106. 10.1055/s-0031-1277005.

[3] Löser C, Fruehauf S, Müller M, Brück P, Hahn L, Lange O, et al. Moderne Ernährungstherapie bei onkologischen Patienten – ein Positionspapier. Aktuel Ernahrungsmed. 2014;39:127–31. 10.1055/s-0034-1369885.

[4] Spiro A, Baldwin C, Patterson A, Thomas J, Andreyev HJN. The views and practice of oncologists towards nutritional support in patients receiving chemotherapy. Br J Cancer. 2006;95:431–4. 10.1038/sj.bjc.6603280. 16880793 10.1038/sj.bjc.6603280 PMC2360668

[5] Kopelman P, Lennard-Jones J. Nutrition and patients: a doctor’s responsibility. Clin Med (Lond). 2002;2:391–4. 10.7861/clinmedicine.2-5-391. 12448582 10.7861/clinmedicine.2-5-391 PMC4953073

[6] Löser C. Nutrition in modern oncology. 1st ed. Bremen: UNI-MED; 2013.

[7] Lucia S, Esposito M, Rossi Fanelli F, Muscaritoli M. Cancer cachexia: from molecular mechanisms to patient’s care. Crit Rev Oncog. 2012;17:315–21. 10.1615/critrevoncog.v17.i3.90. 22831162 10.1615/critrevoncog.v17.i3.90

[8] Fearon K, Strasser F, Anker SD, Bosaeus I, Bruera E, Fainsinger RL, et al. Definition and classification of cancer cachexia: an international consensus. Lancet Oncol. 2011;12:489–95. 10.1016/S1470-2045(10)70218-7. 21296615 10.1016/S1470-2045(10)70218-7

[9] Caburet C, Farigon N, Mulliez A, Mom T, Boirie Y, Gilain L, Saroul N. Impact of nutritional status at the outset of assessment on postoperative complications in head and neck cancer. Eur Ann Otorhinolaryngol Head Neck Dis. 2020;137:393–8. 10.1016/j.anorl.2019.12.005. 31870765 10.1016/j.anorl.2019.12.005

[10] Norman K, Pichard C, Lochs H, Pirlich M. Prognostic impact of disease-related malnutrition. Clin Nutr. 2008;27:5–15. 10.1016/j.clnu.2007.10.007. 18061312 10.1016/j.clnu.2007.10.007

[11] Fearon K, Arends J, Baracos V. Understanding the mechanisms and treatment options in cancer cachexia. Nat Rev Clin Oncol. 2013;10:90–9. 10.1038/nrclinonc.2012.209. 23207794 10.1038/nrclinonc.2012.209

[12] Kang MC, Kim JH, Ryu SW, Moon JY, Park JH, Park JK, et al. Prevalence of malnutrition in hospitalized patients: a multicenter cross-sectional study. J Korean Med Sci. 2018;33:e10. 10.3346/jkms.2018.33.e10. 29215819 10.3346/jkms.2018.33.e10 PMC5729651

[13] Löser C. Malnutrition in hospital: the clinical and economic implications. Dtsch Arztebl Int. 2010;107:911–7. 10.3238/arztebl.2010.0911. 21249138 10.3238/arztebl.2010.0911 PMC3023157

[14] Yoshimura T, Suzuki H, Takayama H, Higashi S, Hirano Y, Tezuka M, et al. Impact of preoperative low prognostic nutritional index and high intramuscular adipose tissue content on outcomes of patients with oral squamous cell carcinoma. Cancers (Basel). 2020. 10.3390/cancers12113167. 33126582 10.3390/cancers12113167 PMC7692578

[15] Tsai Y-T, Lai C-H, Huang T-H, Hsieh C-C, Huang EI, Lee Y-C, et al. Association of malnutrition with postoperative complication risk after curative surgery for oral cancer: Observational study. Med (Baltim). 2020;99:e23860. 10.1097/MD.0000000000023860. 10.1097/MD.0000000000023860 PMC7769301 33350779

[16] Luo K, Chen K, Li Y, Ji Y. Association between sarcopenia and outcomes of surgically treated oral squamous cell carcinoma: a systematic review and meta-analysis. Front Oncol. 2024;14:1445956. 10.3389/fonc.2024.1445956. 39555456 10.3389/fonc.2024.1445956 PMC11564163

[17] Buscemi P, Randazzo C, Buscemi C, Barile AM, Finamore E, Caruso R, et al. Nutritional factors and survival in a cohort of patients with oral cancer. Front Nutr. 2025;12:1530460. 10.3389/fnut.2025.1530460. 40151344 10.3389/fnut.2025.1530460 PMC11948537

[18] Pirlich M, Schwenk A, Müller MJ. DGEM-Leitlinie Enterale Ernährung: Ernährungsstatus. Aktuel Ernahrungsmed. 2003;28:10–25. 10.1055/s-2003-36934.

[19] Wolff, S3-Leitlinie Mundhöhlenkarzinom.: Leitlinienprogramm Onkologie (Deutsche Krebsgesellschaft, Deutsche Krebshilfe, AWMF) (2021): S3-Leitlinie Diagnostik und Therapie des Mundhöhlenkarzinoms, Langversion 3.0, 2021, AWMF-Registernummer: 007/100OL. https://www.leitlinienprogramm-onkologie.de/leitlinien/mundhoehlenkarzinom/ (zuletzt aufgerufen am: 22.08.2021). 2021.

[20] Weir CB, Jan A, StatPearls. BMI classification percentile and cut off points. Treasure Island (FL); 2024.

[21] Cederholm T, Barazzoni R, Austin P, Ballmer P, Biolo G, Bischoff SC, et al. ESPEN guidelines on definitions and terminology of clinical nutrition. Clin Nutr. 2017;36:49–64. 10.1016/j.clnu.2016.09.004. 27642056 10.1016/j.clnu.2016.09.004

[22] Detsky AS, McLaughlin JR, Baker JP, Johnston N, Whittaker S, Mendelson RA, Jeejeebhoy KN. What is subjective global assessment of nutritional status? JPEN J Parenter Enter Nutr. 1987;11:8–13. 10.1177/014860718701100108. 10.1177/014860718701100108 3820522

[23] Kyle UG, Bosaeus I, de Lorenzo AD, Deurenberg P, Elia M, Gómez JM, et al. Bioelectrical impedance analysis—part I: review of principles and methods. Clin Nutr. 2004;23:1226–43. 10.1016/j.clnu.2004.06.004. 15380917 10.1016/j.clnu.2004.06.004

[24] Grundmann O, Yoon SL, Williams JJ. The value of bioelectrical impedance analysis and phase angle in the evaluation of malnutrition and quality of life in cancer patients—a comprehensive review. Eur J Clin Nutr. 2015;69:1290–7. 10.1038/ejcn.2015.126. 26220573 10.1038/ejcn.2015.126

[25] Małecka-Massalska T, Smoleń A, Morshed K. Extracellular-to-body cell mass ratio and subjective global assessment in head-and-neck cancers. Curr Oncol. 2014;21:e62–6. 10.3747/co.21.1671. 24523622 10.3747/co.21.1671 PMC3921049

[26] Büntzel J, Krauß T, Büntzel H, Küttner K, Fröhlich D, Oehler W, et al. Nutritional parameters for patients with head and neck cancer. Anticancer Res. 2012;32:2119–23. 22593498

[27] Schütz T, Plauth M. Subjective global assessment - a method for the assessment of nutritional state. Akt Ernähr Med. 2005;30:43–8. 10.1055/s-2004-834559.

[28] Barbosa-Silva MCG, Barros AJD, Post CLA, Waitzberg DL, Heymsfield SB. Can bioelectrical impedance analysis identify malnutrition in preoperative nutrition assessment? Nutrition. 2003;19:422–6. 10.1016/s0899-9007(02)00932-2. 12714094 10.1016/s0899-9007(02)00932-2

[29] Irgebay Z, Beiriger JC, Beiriger JW, Matinrazm S, Natali M, Yi C, et al. Review of diet protocols following orthognathic surgery and analysis of postoperative weight loss. Cleft Palate Craniofac J. 2023;60:1411–8. 10.1177/10556656221113998. 35837697 10.1177/10556656221113998

[30] Ooi K, Inoue N, Matsushita K, Yamaguchi H, Mikoya T, Kawashiri S, Tei K. Body weight loss after orthognathic surgery: comparison between postoperative intermaxillary fixation with metal wire and elastic traction, factors related to body weight loss. J Maxillofac Oral Surg. 2021;20:95–9. 10.1007/s12663-019-01318-6. 33584049 10.1007/s12663-019-01318-6 PMC7855110

[31] Inaba Y, Hasebe D, Hashizume K, Suda D, Saito N, Saito D, et al. Changes in nutritional status of patients with jaw deformities due to orthognathic surgery. Oral Surg Oral Med Oral Pathol Oral Radiol. 2023;135:347–54. 10.1016/j.oooo.2022.07.007. 36244953 10.1016/j.oooo.2022.07.007

